# SNAP Emergency Allotments, Emergency Rent Assistance, Rent Burden, and Housing and Food Security, June 2022–May 2023

**DOI:** 10.5888/pcd21.240121

**Published:** 2024-08-29

**Authors:** Patrick J. Brady, Kaitlyn M. Berry, Rachel Widome, Sruthi Valluri, Melissa N. Laska

**Affiliations:** 1 Division of Epidemiology and Community Health, University of Minnesota School of Public Health, Minneapolis, Minnesota; 2 Brigham and Women’s Hospital, Boston, Massachusetts

## Abstract

**Introduction:**

During the COVID-19 pandemic, Supplemental Nutrition Assistance Program (SNAP) emergency allotments and emergency rent assistance provided support to low-income households. Rent burden, a form of housing insecurity, can severely limit household resources, which, in turn, affects health equity. We explored whether these policy interventions equitably supported households that were or were not experiencing rent burden.

**Methods:**

We used data from the US Household Pulse Survey (June 2022–May 2023) to examine whether associations between emergency support policies and indicators of food and housing security differed according to household rent burden status. We modeled each outcome (food sufficiency or being current on rent) as a function of policy exposure (SNAP emergency allotments or emergency rent assistance), rent burden, and their interaction. We included demographic characteristics, state of residence, and survey cycle as covariates. We modeled each outcome and policy exposure combination separately.

**Results:**

Receiving emergency allotments (72.4% vs 67.2% for SNAP participants in states with and without emergency allotments, respectively) and emergency rent assistance (64.5% vs 57.6% for households that received and were waitlisted/denied assistance, respectively) were associated with greater food sufficiency. The relationship between emergency allotments and food sufficiency was stronger in rent-burdened households; however, emergency rent assistance supported food sufficiency to a greater extent in non–rent-burdened households. Emergency rent assistance supported households in being current on rent (78.7% vs 56.4% for households that received and were waitlisted/denied assistance, respectively) and supported being current on rent to a greater extent in non–rent-burdened households than in rent-burdened households.

**Conclusion:**

The relationship between emergency support policies and food or housing security differed according to whether households were experiencing rent burden. Associations were sometimes stronger in less economically constrained conditions. These results indicate an opportunity to better design policies to support low-income households, address food and housing security, and ultimately decrease the prevalence of chronic disease.

**Summary**

**What is already known on this topic?**

Economic supports implemented in the US during the COVID-19 pandemic improved material conditions in US households, but we do not know if improvements differed according to a household’s economic situation.

**What is added by this report?**

The association between receiving Supplemental Nutrition Assistance Program emergency allotments or emergency rent assistance and indicators of food or housing security differed according to whether households were experiencing rent burden, sometimes with stronger, positive associations for non–rent-burdened households.

**What are the implications for public health practice?**

Policies that support public health and prevent chronic disease by addressing social determinants of health should be purposefully designed to equitably address the needs of economically constrained households.

## Introduction

The COVID-19 pandemic exposed the inadequacy of the US social safety net. However, a robust policy response supported households through many mechanisms, including direct economic support (eg, enhanced child tax credit, economic impact payments, enhanced unemployment insurance), a wider food safety net (increases in Supplemental Nutrition Assistance Program [SNAP] benefits, SNAP emergency allotments, and Pandemic EBT [electronic benefits transfer] to replace school meals), and an enhanced housing safety net (emergency rental assistance, eviction moratoriums) (1–8). A growing body of research on the policy response to the pandemic provides evidence that state provision of economic support to low-income households improves their material security (2–8). Policies that meet economic needs are therefore a major part of the solution to the problems of material insecurities that drive health inequities in preventable chronic diseases.

Food security, or “access by all people at all times to enough food for an active, healthy life” (9), is recognized as a critical social determinant of health, and lack of access to adequate food is a key contributor to the prevalence of and disparities in chronic disease (10,11). Inadequate food access drives risk of and disparities in nutrition-related chronic disease outcomes, such as for cardiovascular diseases and type 2 diabetes (10,11). Substantial evidence indicates that SNAP, which provides in-kind support to purchase eligible foods from authorized retailers, supports food security (12). SNAP emergency allotments were enacted in 2020 as a temporary measure that provided the maximum benefit amount for SNAP participants during the COVID-19 public health emergency (13). Before April 2021, households already receiving the maximum allowable amount, the most economically constrained participants, were excluded from receiving additional support (13). Beginning in April 2021, SNAP emergency allotments provided at least an additional $95 per household per month for households receiving the maximum allowable amount (3,4,13). This revised policy ensured that all SNAP participants in states implementing emergency allotments received additional benefits (13). Eighteen states chose to end SNAP emergency allotments early before the program expired in February 2023. Ending emergency allotments reduced benefits by an estimated average of $90 per person per month (13) and increased the number of households experiencing food insufficiency in the previous week from 25% to 31% (3).

Another key social determinant of health, housing security, is the “availability of and access to stable, safe, adequate, and affordable housing and neighborhoods regardless of gender, race, ethnicity, or sexual orientation” (14). During the COVID-19 pandemic, approximately 16% of households were behind on their rent, and this disproportionality affected racially and ethnically minoritized households (15). A critical dimension of housing security is cost burden, or the amount of income put toward housing costs (14,16,17). Renters who spend more than 30% of their income on rent are considered rent burdened (17,18). Rent burden is increasing in the US and is now recognized as a pressing public health issue (16,19). Housing unaffordability and rent burden are associated with negative social and economic outcomes (20), including food insecurity (21–24). Housing insecurity drives chronic disease outcomes and disparities such as food insecurity (11) by determining the food and health environment in which a household exists and by contributing to resource constraint. Addressing housing insecurity may support food security (25–27). One intervention to reduce housing insecurity is emergency rent assistance, which often directly covers unpaid rent owed to the landlord and/or unpaid utility costs. Many new state and local emergency rent assistance programs emerged early in 2020 and throughout the pandemic (7). These programs were often funded through federal legislation (28,29) and represent the first widescale implementation of this kind of housing assistance. Emergency rent assistance has been shown to reduce eviction filings (5), decrease the financial burden associated with housing insecurity (6,8), and improve mental health (6,8). Despite the beneficial effects of emergency rent assistance (5–8), these programs encountered implementation challenges and were not designed to address long-standing, systemic issues around housing affordability (30–32).

Generally, we know that SNAP emergency allotments (3,4) and emergency rent assistance (5–8) had positive effects. These effects, however, may have differed according to the material context of households. The objective of our study was to examine whether the association between receiving SNAP emergency allotments or emergency rent assistance and indicators of food and housing security differed according to whether households were experiencing rent burden. Based on previous literature (3,4,6,8,18), we hypothesized that experiencing rent burden would decrease food and housing security among low-income renters, whereas receiving SNAP emergency allotments or receiving emergency rent assistance would increase food and housing security. We also hypothesized that a significant interaction would exist between experiencing rent burden and each policy exposure. We did not hypothesize the direction of the interaction effect, evaluating it only in an exploratory manner.

## Methods

We used data from the nationally representative US Census Bureau Household Pulse Survey (HPS) from June 2022 through May 2023, which included cycles 46 to 57. We selected these cycles because of the availability of the variable (monthly rental cost) needed to evaluate experiences of rent burden. More details on the HPS methodology are published elsewhere (33). Briefly, the HPS is a repeated cross-sectional survey designed to assess household social and economic conditions. Households are sampled from the US Census Bureau’s Master Address File, and information from the US Census Bureau’s Contact Frame enables contact with respondents through email and text messages. A single respondent answers questions about their household. Survey cycles typically lasted about 2 weeks. For the cycles used in our analysis, a gap of approximately 2 weeks occurred between each cycle. Survey weights account for survey design and nonresponse to generate demographically representative national-level estimates based on educational attainment, sex, age, race, and ethnicity.

### Sample

Our sample consisted of renters with incomes less than 130% of the federal poverty threshold (N = 40,895). We used the self-reported categorical measure of yearly income and household size (number of household members) to determine eligibility based on income. If a household in any income category was under 130% of the federal poverty threshold, we included that household in the under-130% category. For example, the federal poverty threshold for a 3-member household is $29,939. Thus, a 3-member household with a yearly income of either less than $25,000 or $25,000 to $34,999 would be included in the under-130% category. Likewise, households with a yearly income of $50,000 to $74,999 would need to include at least 7 members to be considered a low-income renter for the purposes of our study.

### Measures

Our outcomes of interest were food sufficiency and being current on rent. Food sufficiency, a dimension of food security, describes households with enough food for all household members (10). We assessed food sufficiency with a single item that asked households about the amount and types of foods they had available to eat in the past week. Households were considered “food sufficient” if they had enough of the kind of foods or enough but not always the kinds of food they wanted to eat; “food insufficient” described households that sometimes or often did not have enough to eat. Being unable to make rent payments is a housing affordability dimension of housing insecurity and is a more severe measure of housing affordability than experiencing rent burden (14). Being current on rent payments was assessed with a single item that asked, “Is this household currently caught up on rent payments?” Response options were yes or no.

We were interested in exposure to 2 policies: SNAP emergency allotments and emergency rent assistance. Because some states discontinued SNAP emergency allotments before the program expired in February 2023, households that were exposed to this policy can be identified according to participation, state of residence, and time period (3,4). We used indicator variables for exposure to emergency allotments based on self-reported SNAP participation at the time of the survey, state of residence, and survey cycle in a similar manner to previously reported analyses (3,4). This process resulted in a 4-level variable: 1) SNAP participant in a state with emergency allotments, 2) SNAP participant in a state without emergency allotments, 3) non–SNAP participant in a state with emergency allotments, and 4) non–SNAP participant in a state without emergency allotments. Our indicator for emergency rent assistance was based on the self-reported answer to the question “Have you or anyone in your household applied for emergency rental assistance through your state or local government to cover your unpaid rent or utility bills?” Response options were “Yes, received emergency rent assistance,” “No, waitlisted or denied emergency rent assistance,” or “No, did not apply.” Our moderator was experiencing rent burden (yes or no). We coded a household as experiencing rent burden if yearly rent costs (calculated from current monthly rent at the time of the survey) divided by the midpoint of income category was 0.30 or greater.

We controlled for demographic characteristics known to be associated with socioeconomic status. These were age category of respondent (≤35, 36–64, or ≥65 y), presence of children in household (yes or no), gender identity of respondent (male, female, transgender or other than any listed option), racial self-classification of respondent (Asian, Black, White, or any other race alone or any combination of responses), self-classification of respondent as having Hispanic, Latino, or Spanish origin (yes or no), yearly household income (<$25,000, $25,000–$34,999, $35,000–$49,999, or $50,000–$74,999), if anyone in household experienced a job loss in the past 4 weeks (yes or no), if anyone in household had employment in the past week (yes or no), educational attainment of respondent (less than high school or some high school, high school graduate or equivalent, some college but degree not received or in progress, associate or bachelor’s degree, graduate degree), marital status (now married, widowed, divorced, separated, never married), and if a household received food aid. The yes–no question about food aid asked if, during the last 7 days, anyone in the household got free groceries from a food pantry, food bank, church, or other place that helps with free food. Gender identity, racial self-classification, and Hispanic self-classification variables were included as proxies for experiences of structural and systemic discrimination that affect food, housing, and economic security. Finally, we included fixed effects for state (each of the 50 states and the District of Columbia) and the survey cycle.

### Analysis

First, we produced descriptive statistics for all variables overall and stratified by policy exposure. We also descriptively examined our outcomes during the study period. Then, we estimated the main effects of each policy exposure and the moderator on our outcomes of interest. Specifically, we used logistic regressions to model the outcomes (household food sufficiency or being current on rent) as a function of receiving SNAP emergency allotments, receiving emergency rent assistance, or experiencing rent burden. We adjusted models for demographic characteristics, state, and survey cycle. We tested separately the effect of each exposure or moderator on each outcome, resulting in 6 main effect regression models. From each model, we calculated the predicted probability of experiencing the outcome for each level of the exposure–moderator variable. We then calculated the difference in the predicted probability of the outcome across exposure–moderator levels.

We then ran interaction models to test whether the effect of each policy exposure differed according to whether households were rent burdened. Specifically, we estimated the outcomes (food sufficiency or being current on rent) as a function of receiving SNAP emergency allotments or emergency rent assistance, experiencing rent burden, and the interaction between policy exposure and rent burden. Each exposure–moderator interaction was tested separately. As with the main effects models, we then calculated the predicted probability of experiencing the outcome for each level of the interaction. We then calculated the differences in predicted probabilities for key comparisons of interest, using contrast statements following the interaction model.

Finally, we wanted to examine the effect of the decision to use the midpoint of each income category when determining whether a household experienced rent burden. Therefore, we conducted a sensitivity analysis where a household was designated rent burdened if their yearly rent costs divided by the top level of their income category was 0.30 or greater (ie, assuming each household had the highest possible income for their income category instead of the midpoint). We used Stata version 17 (StataCorp LLC) for all analyses. All analyses were weighted as described in HPS technical documentation (33).

## Results

In the overall sample, 83.4% reported being rent burdened, 69.2% food sufficient, and 80.4% current on rent (Table 1). Overall, 63.7% were White, 23.8% were Black, and 24.2% were of Hispanic, Latino, or Spanish origin; 62.4% of respondents identified as female. The annual household income category with the largest percentage (70.6% of respondents) was less than $25,000; 41.6% had at least a high school diploma, and 17.7% received food aid. The mean (SD) household size was 3.0 (1.5) members. The rates of households experiencing food sufficiency and being current on rent were relatively consistent during the study period, ranging from 65.6% to 72.4% (food sufficiency) and 76.8% to 83.0% (current on rent) (Figure).

**Table 1:** Demographic Characteristics of Low-Income Renters Included in Analytic Sample From Analysis of US Census Bureau Household Pulse Survey Releases 46–57, June 2022–May 2023^a^

Characteristic	Overall sample (N = 40,895)^b^	Receipt of SNAP emergency allotments (EAs)				Receipt of emergency rent assistance		
		SNAP participant in a state with EAs (n = 9,874)	SNAP participant in a state without EAs (n = 7,997)	Non–SNAP participant in a state with EAs (n = 11,401)	Non–SNAP participant in a state without EAs (n = 11,623)	Yes (n = 7,712)	No, waitlisted or denied (n = 3,886)	No, did not apply (n = 29,297)
**Rent burdened (spend >30% of income on rent)**								
Yes	83.4	79.9	78.7	87.8	85.5	75.2	89.3	84.5
No	16.6	20.1	21.3	12.2	14.5	24.8	10.7	15.5
**Food sufficiency (had enough of the kind of foods or enough but not always the kinds of food they wanted to eat in the past week)**								
Food sufficient	69.2	69.1	64.6	70.8	71.0	61.7	52.2	73.3
Food insufficient	30.8	30.9	35.4	29.2	29.0	38.3	47.8	26.7
**Current on rent**								
Yes	80.4	77.3	77.0	82.6	83.9	77.1	48.2	85.7
No	19.6	22.7	23.0	17.4	16.1	22.9	51.8	14.3
**Received SNAP EAs**								
SNAP participant in a state with EAs	27.2	100.0	—	—	—	43.3	34.8	22.6
SNAP participant in a state without EAs	18.3	—	100.0	—	—	40.1	24.1	14.8
Non–SNAP participant in a state with EAs	30.6	—	—	100.0	—	14.3	23.8	35.3
Non–SNAP participant in a state without EAs	23.8	—	—	—	100.0	12.3	17.3	27.3
**Received rent assistance^c^**								
Yes	16.5	26.3	27.2	7.7	8.5	100.0	—	—
No, waitlisted or denied	10.3	13.1	13.5	8.0	7.5	—	100.0	—
No, did not apply	73.2	60.6	59.3	84.3	84.0	—	—	100.0
**Age, y**								
≤35	35.5	28.1	29.3	41.5	41.0	26.2	35.7	37.5
36–64	48.1	54.8	53.4	43.6	42.1	56.9	55.2	45.1
≥65	16.4	17.1	17.3	14.9	16.9	16.9	9.1	17.4
**Children in household**								
Yes	53.8	51.3	53.2	40.8	41.8	50.0	58.1	43.7
No	46.2	48.7	46.8	59.2	58.2	50.0	41.9	56.3
**Gender identity**								
Male	34.2	27.6	26.3	41.0	38.9	26.3	30.3	36.5
Female	62.4	69.1	71.4	55.1	57.5	70.9	66.4	60.0
Transgender or other than any listed option	3.4	3.3	2.3	3.9	3.6	2.8	3.3	3.5
**Race**								
Asian	3.7	3.1	2.0	5.4	3.6	1.8	2.2	4.3
Black	23.8	30.0	31.7	17.1	19.5	32.7	36.6	20.1
White	63.7	57.2	57.9	68.3	69.6	55.7	48.6	67.6
Any other single race or any combination	8.8	9.7	8.4	9.2	7.3	9.8	12.6	8.0
**Hispanic, Latino, or Spanish origin**								
Yes	24.2	23.3	18.9	29.8	22.3	20.1	24.9	25.0
No	75.8	76.7	81.1	70.2	77.7	79.9	75.1	75.0
**Annual household income, $**								
<25,000	70.6	79.1	78.1	64.0	63.4	79.4	69.2	68.8
25,000–34,999	18.4	14.1	15.0	21.9	21.7	13.5	19.4	19.4
35,000–49,999	10.2	6.1	6.2	13.2	13.9	6.7	11.1	10.8
50,000–74,999	0.8	0.7	0.7	0.9	1.0	0.4	0.3	1.0
**Anyone in household experienced a job loss in the past 4 weeks**								
Yes	21.8	22.7	22.6	22.2	19.7	23.1	38.7	19.1
No	78.2	77.3	77.4	77.8	80.3	76.9	61.3	80.9
**Anyone in household had employment in the past week**								
Yes	44.3	30.5	32.0	56.0	54.7	32.2	44.0	47.1
No	55.7	69.5	68.0	44.0	45.3	67.8	56.0	52.9
**Educational attainment**								
Less than high school or some high school	16.8	21.1	19.0	15.7	11.5	18.2	19.4	16.1
High school graduate or equivalent	41.6	43.2	45.2	38.1	41.3	41.5	40.2	41.8
Some college, but degree not received or in progress	22.9	21.6	21.8	23.5	24.6	24.0	23.9	22.5
Associate or bachelor’s degree	15.4	12.4	11.9	17.9	18.4	14.0	14.2	15.9
Graduate degree	3.3	1.7	2.1	4.8	4.2	2.3	2.3	3.7
**Received food aid^d^**								
Yes	17.7	22.0	25.5	13.4	12.2	26.6	23.5	14.8
No	82.3	78.0	74.5	86.6	87.8	73.4	76.5	85.2
**Marital status**								
Now married	22.5	20.6	19.5	24.7	24.0	19.3	23.3	23.0
Widowed	6.4	6.8	6.4	5.6	7.1	7.2	4.9	6.5
Divorced	21.3	24.0	25.4	17.2	20.4	25.2	19.7	20.7
Separated	6.0	6.8	6.9	5.8	4.6	7.2	6.6	5.6
Never married	43.8	41.8	41.8	46.7	43.9	41.1	45.5	44.2
**No. of household members, mean (SD)**	3.0 (1.5)	3.1 (1.5)	3.2 (1.7)	3.0 (1.4)	2.9 (1.6)	2.9 (1.5)	3.4 (1.5)	3.0 (1.5)

Abbreviations: — , does not apply; EA, emergency allotment; SNAP, Supplemental Nutrition Assistance Program.

a Low-income renters defined as renters with household incomes <130% of the federal poverty threshold. All values are percentages unless otherwise indicated.

b Percentages may not sum to 100 because of rounding.

c Survey question was, “Have you or anyone in your household applied for emergency rental assistance through your state or local government to cover your unpaid rent or utility bills?”

d Survey question was, “During the last 7 days, did you or anyone in your household get free groceries from a food pantry, food bank, church, or other place that helps with free food?” Response options were yes or no.

**Figure:**
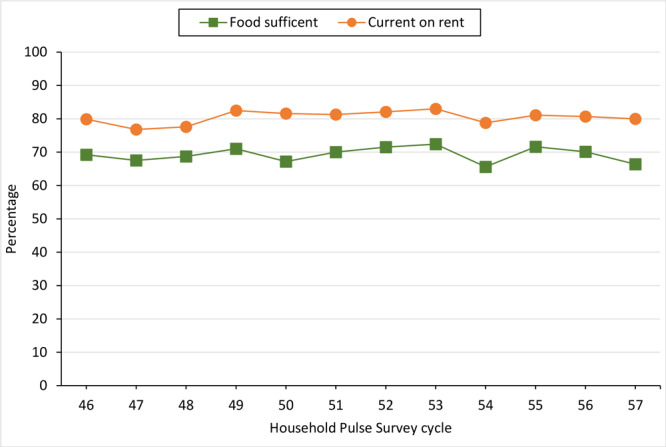
Weighted percentage of respondents who reported being food sufficient and current on rent payments, by survey cycle, US Census Bureau Household Pulse Survey Releases 46–57, June 2022–May 2023.

**Table:** 

Survey cycle	Food sufficient	Current on rent
46	69.2	79.9
47	67.5	76.8
48	68.7	77.6
49	71.0	82.5
50	67.2	81.6
51	70.0	81.3
52	71.5	82.1
53	72.4	83.0
54	65.6	78.8
55	71.6	81.1
56	70.1	80.7
57	66.4	80.0

### Food sufficiency

In the main effects models, food sufficiency was less common among households that experienced rent burden than among those that did not (68.7% vs 71.7%), higher among SNAP participants who received emergency allotments than among those that did not (72.4% vs 67.2%), and higher among households that received emergency rent assistance than among those who were waitlisted or denied (64.5% vs 57.6%) (Table 2). In the interaction model for SNAP emergency allotments and rent burden, emergency allotments supported food sufficiency when SNAP participating households were rent burdened (72.0% vs 66.0%) but not if they were non–rent burdened (74.0% vs 71.5%) (Table 2). In the interaction model for emergency rent assistance and rent burden, receiving assistance supported food sufficiency to a greater extent when households who applied for assistance were non–rent burdened (68.9% vs 55.9% for non–rent-burdened households and 63.0% vs 57.8% for rent-burdened households) (Table 2).

**Table 2:** Predicted Probability of Experiencing Food Sufficiency Among Low-Income Renters (N = 40,895), Based on Rent-Burden Status and Receipt of Pandemic-Related Economic Supports, Estimated From Main Effects and Interaction Models From Analysis of US Census Bureau Household Pulse Survey Releases 46–57, June 2022–May 2023^a^

Exposure	No. of survey respondents	Predicted probability of food sufficiency, % (95% CI)	Difference in predicted probabilities, percentage point (95% CI)
**Main effect models**			
**Rent burdened (spend >30% of income on rent)**			
Yes	33,959	68.7 (68.0 to 69.5)	−3.0 (−4.8 to −1.1)
No	6,936	71.7 (69.9 to 73.4)	Reference
**Receipt of SNAP EAs**			
SNAP participant in state with EAs	9,874	72.4 (70.3 to 74.5)	5.2 (1.7 to 8.7)
SNAP participant in state without EAs	7,997	67.2 (65.2 to 69.2)	Reference
Non–SNAP participant in state with EAs	11,401	68.8 (66.8 to 70.8)	1.6 (−1.8 to 5.0)
Non–SNAP participant in state without EAs	11,623	67.4 (65.2 to 69.7)	0.2 (−1.8 to 2.3)
**Receipt of ERA^b^**			
Yes	7,712	64.5 (62.8 to 66.2)	6.9 (3.9 to 9.8)
No, waitlisted or denied ERA	3,886	57.6 (55.1 to 60.2)	Reference
No, did not apply	29,297	72.1 (71.3 to 72.9)	14.5 (11.8 to 17.2)
**Interaction models**			
**Rent burdened × Receipt of SNAP EAs**			
**Rent burdened **			
SNAP participant, state with EAs	7,877	72.0 (69.7 to 74.3)	5.9 (2.0 to 9.8)
SNAP participant, state without EAs	6,169	66.0 (63.7 to 68.4)	Reference
Non–SNAP participant, state with EAs	10,044	68.3 (66.2 to 70.5)	2.3 (−1.5 to 6.1)
Non–SNAP participant, state without EAs	9,869	67.6 (65.2 to 70.0)	1.6 (−0.9 to 4.1)
**Non–rent burdened**			
SNAP participant, state with EAs	1,997	74.0 (70.7 to 77.3)	2.5 (−2.0 to 7.0)
SNAP participant, state without EAs	1,828	71.5 (68.7 to 74.3)	Reference
Non–SNAP participant, state with EAs	1,357	72.1 (67.9 to 76.3)	0.6 (−4.3 to 5.5)
Non–SNAP participant, state without EAs	1,754	66.8 (63.3 to 70.2)	−4.7 (−8.6 to −0.8)
**Rent-burdened × Receipt of ERA**			
**Rent burdened **			
Yes, received ERA	5,893	63.0 (61.0 to 65.1)	5.2 (2.0 to 8.5)
No, waitlisted or denied ERA	3,412	57.8 (55.1 to 60.5)	Reference
No, did not apply	24,654	71.7 (70.8 to 72.6)	13.9 (10.9 to 16.9)
**Non–rent burdened **			
Yes, received ERA	1,819	68.9 (65.3 to 72.6)	13.0 (5.1 to 20.9)
No, waitlisted or denied ERA	474	55.9 (49.0 to 62.8)	Reference
No, did not apply	4,643	74.5 (72.4 to 76.6)	18.5 (11.5 to 25.6)

Abbreviations: EA, emergency allotment; ERA, emergency rent assistance; SNAP, Supplemental Nutrition Assistance Program.

a Low-income renters defined as renters with household incomes <130% of the federal poverty threshold. Main effect and interaction models were estimated separately for each exposure/interaction; all models adjusted for demographic characteristics with fixed effects for state and survey release.

b Survey question was, “Have you or anyone in your household applied for emergency rental assistance through your state or local government to cover your unpaid rent or utility bills?”

### Current on rent

In the main effects models, being current on rent was less common among households that experienced rent burden than among non–rent-burdened households (79.5% vs 85.1%) (Table 3). The predicted probability of being current on rent was similar across SNAP participants and non–SNAP participants regardless of policy exposure. Among households that applied for emergency rent assistance, those who received assistance (78.7%) were substantially more likely to be current on rent than those who were waitlisted or denied (56.4%) (Table 3). In the interaction model for SNAP emergency allotments and rent burden, we found no clear relationship between experiencing rent burden and being exposed to SNAP emergency allotments. In the interaction model for emergency rent assistance and rent burden, assistance supported being current on rent to a greater extent when households were non–rent burdened (85.5% vs 60.9% for non–rent-burdened households and 76.7% vs 55.8% for rent-burdened households) (Table 3).

**Table 3:** Predicted Probability of Being Behind on Rent Among Low-Income Renters (N = 40,895), by Rent-Burdened Status and Receipt of Pandemic-Related Economic Supports, US Census Bureau Household Pulse Survey Releases 46–57, June 2022–May 2023^a^

Exposure	No. of survey respondents	Predicted probability of being current on rent, % (95% CI)	Difference in predicted probabilities, percentage point (95% CI)
**Main effect models**			
**Rent burdened (spend >30% of income on rent)**			
Yes	33,959	79.5 (78.6 to 80.4)	−5.6 (−7.3 to −3.9)
No	6,936	85.1 (83.6 to 86.6)	Reference
**SNAP EAs**			
SNAP participant, state with EAs	9,874	79.6 (77.9 to 81.3)	1.0 (−2.6 to 4.6)
SNAP participant, state without EAs	7,997	78.6 (76.1 to 81.0)	Reference
Non–SNAP participant, state with EAs	11,401	81.7 (80.3 to 83.2)	3.2 (−0.2 to 6.5)
Non–SNAP participant, state without EAs	11,623	81.4 (79.3 to 83.5)	2.8 (0.5 to 5.2)
**Receipt of ERA^b^**			
Yes, received ERA	7,712	78.7 (76.9 to 80.6)	22.4 (18.8 to 25.9)
No, waitlisted or denied ERA	3,886	56.4 (53.5 to 59.3)	Reference
No, did not apply	29,297	84.7 (84.0 to 85.5)	28.3 (25.2 to 31.4)
**Interaction models**			
**Rent burdened × receipt of SNAP EAs**			
**Rent burdened **			
SNAP participant, state with EAs	7,877	78.3 (76.5 to 80.1)	2.0 (−1.8 to 5.8)
SNAP participant, state without EAs	6,169	76.3 (73.7 to 78.9)	Reference
Non–SNAP participant, state with EAs	10,044	81.1 (79.6 to 82.7)	4.8 (1.3 to 8.3)
Non–SNAP participant, state without EAs	9,869	81.4 (79.2 to 83.5)	5.1 (2.6 to 7.6)
**Non–rent burdened **			
SNAP participant, state with EAs	1,997	84.5 (81.8 to 87.3)	−3.1 (−7.1 to 1.0)
SNAP participant, state without EAs	1,828	87.6 (84.7 to 90.6)	Reference
Non–SNAP participant, state with EAs	1,357	85.4 (82.4 to 88.5)	−2.2 (−6.9 to 2.5)
Non–SNAP participant, state without EAs	1,754	82.7 (76.9 to 85.9)	−4.9 (−8.8 to −1.0)
**Rent burdened × receipt of ERA**			
**Rent burdened**			
Yes, received ERA	5,893	76.7 (74.4 to 78.9)	20.9 (16.9 to 24.8)
No, waitlisted/denied ERA	3,412	55.8 (52.7 to 58.8)	Reference
No, did not apply	24,654	84.1 (83.3 to 85.0)	28.3 (25.1 to 31.5)
**Non–rent burdened**			
Yes, received ERA	1,819	85.5 (83.3 to 87.8)	24.8 (17.5 to 32.0)
No, waitlisted or denied ERA	474	60.9 (53.7 to 62.8)	Reference
No, did not apply	4,643	87.9 (86.3 to 89.5)	27.1 (19.5 to 34.7)

Abbreviations: EA, emergency allotment; ERA, emergency rent assistance; SNAP, Supplemental Nutrition Assistance Program.

a Low-income renters defined as renters with household incomes <130% of the federal poverty threshold. Main effect and interaction models were estimated separately for each exposure/interaction; all models adjusted for demographic characteristics with fixed effects for state and survey release.

b Survey question was, “Have you or anyone in your household applied for emergency rental assistance through your state or local government to cover your unpaid rent or utility bills?”

### Sensitivity analysis

In the sensitivity analysis, assuming that each household had the highest possible income for their income category, instead of the midpoint, the main effect of rent burden was diminished, but trends in the interaction models were similar.

## Discussion

We found that while both SNAP emergency allotments and emergency rent assistance supported household economic security, the association between receiving these benefits and food or housing security was affected by whether the household was rent burdened. Receiving SNAP emergency allotments was more strongly associated with food sufficiency in rent-burdened households than in non–rent-burdened households. In contrast, emergency rent assistance was more strongly associated with food sufficiency and being current on rent in non–rent-burdened households than in rent-burdened households. Together, these findings suggest 2 possible mechanisms for the interaction between economic supports and experiencing rent burden. Programs that provide in-kind support may have a stronger effect in more economically constrained households because the resources will have a larger relative contribution to their financial capabilities. At the same time, households with fewer material constraints may be able to use additional resources more effectively to meet their needs. Future research, especially mixed-methods studies that can quantitatively assess resource allocation and qualitatively characterize participant experiences and perceptions driving those decisions, should examine these mechanisms.

Consistent with previous research (3,4), in our analysis SNAP emergency allotments supported the food sufficiency of households. We extended this research by documenting the differential effects of household rent burden. That emergency allotments had more effect among rent-burdened households than among non–rent-burdened households provides further support that increasing SNAP benefits could be a key component in supporting food security. These results also support a need to examine how policies interact with social, political, and economic marginalization to produce inequities and potentially drive health disparities. Future research should examine how the effects described here vary by demographic characteristics (eg, racial and ethnic identity, gender identity, household composition) and environmental contexts (eg, housing markets, food environments); whether the observed relationships are consistent across other policies that provide economic support; and how these contribute to a disparate burden of chronic disease on economically marginalized populations.

Although our analysis adds to the evidence on the effects of SNAP emergency allotments, these allotments were designed as a temporary measure. Providing the full benefit amount to all SNAP-participating households may not be a feasible long-term solution, and other mechanisms that increase benefits should be explored. A policy lever that may be of particular interest for simultaneously addressing food and housing security is the SNAP excess shelter deduction (34), which allows households applying for SNAP to claim a federally capped portion of their housing-related costs when determining net income. This deduction affects both eligibility and benefits: eligibility for SNAP is determined by net income test (at or below 100% of the federal poverty threshold) and net income is used in benefits computation (34). The excess shelter deduction could be more actively promoted to increase awareness or improved by removing administrative burdens to apply or increasing associated benefits. Some efforts to improve the excess shelter deduction have been proposed; the Closing the Meal Gap Act (35) would eliminate the deduction’s cap.

Our study results also have important implications for building on the success of emergency rental assistance programs implemented during the COVID-19 pandemic. Our results show that pandemic-related emergency rent assistance programs supported the housing security of non–rent-burdened households to a greater extent than it supported rent-burdened households. This finding could be due to assistance being more effective at addressing economic outcomes among non–rent-burdened households, which are likely less resource-constrained than rent-burdened households. Relatedly, rent-burdened households may be allocating any freed-up resources to more pressing needs, such as transportation, health care and prescription drugs, or childcare, rather than housing. Further research examining how households receiving emergency rent assistance allocate their resources before and after receiving benefits could provide more insight into these mechanisms. Such research could then be used to enhance rental assistance program design (eg, reaching those who are in most need or linking with other forms of support).

We also found that while emergency rent assistance supported both food and housing security, SNAP emergency allotments supported only food security, perhaps because of the relatively meager benefits offered by SNAP overall and, by extension, emergency allotments. Previous research showed that housing cost assistance can improve food security (25–27), but more research is needed to examine the effect, if any, of food assistance on housing security.

Overall, about 3 in 10 households in our analysis did not have enough food sometimes or often in the past week and nearly 20% were not current on rent payments. Additionally, even among non–rent-burdened households, a substantial number of households were facing housing insecurity and inadequate food access, highlighting the need for a more robust social safety net. Without addressing these social determinants of health, it will be impossible to reduce the incidence of preventable chronic diseases and reduce or eliminate disparities between populations. In a country with adequate resources to feed and house all, it is a societal failing that so many struggle with meeting their basic needs. Improving the social safety net, including supporting and advocating for a robust welfare state, should be a priority and emphasis should be placed on including those most affected by socially and politically manufactured food and housing scarcity (36).

### Limitations

Our study has several potential limitations. We used specific dependent variables to reflect wide, multidimensional states of food and housing security. Additionally, our moderator was a measure of rent burden based on the percentage of income needed to pay rent, which does not fully capture the economic reality facing households. While other measures of rent burden, such as the residual income approach (17), more accurately reflect household resources, these measures require more detailed information on income and costs and therefore were not feasible for our analysis. Furthermore, because income data were reported categorically, we were unable to precisely identify households with incomes under 130% of the federal poverty threshold, and we calculated our moderator by using the midpoint of income categories, which may have misclassified some households. Additionally, we relied on self-report to establish receipt of benefits, which likely misclassified some households, with underreporting of receipt more likely than overreporting. There is also substantial selection into assistance programs based on a plethora of observable and unobservable factors, as demonstrated extensively in SNAP (12), for which our analysis did not account. This factor should be considered when comparing the relationship between SNAP participants and non-SNAP participants and households that applied for emergency rental assistance versus those that did not. Finally, our analysis focused on the moderating effect of rent burden and policies that economically supported households on indicators of food and housing security, but more advanced methods, such as difference-in-difference models, could be used to evaluate these relationships more fully.

### Conclusion

The association between receiving economic support and outcomes related to food and housing security differs according to the rent-burdened status of households. This association did not always occur consistently: the association was stronger among rent-burdened households (vs non–rent-burdened households) for SNAP emergency allotments but stronger among non–rent-burdened households (vs rent-burdened households) for emergency rent assistance. Policies and programs that aim to address insecurity, such as food and housing assistance programs, should be designed to provide additional resources to households with fewer resources if those programs are shown to be more effective among less economically constrained households. Such designs could better address the social and economic conditions faced by resource-limited households and contribute to lessening the effect of economic inequities on chronic disease burden and disparities.
